# Attention-Based Models for Classifying Small Data Sets Using Community-Engaged Research Protocols: Classification System Development and Validation Pilot Study

**DOI:** 10.2196/32460

**Published:** 2022-09-06

**Authors:** Brian J Ferrell, Sarah E Raskin, Emily B Zimmerman, David H Timberline, Bridget T McInnes, Alex H Krist

**Affiliations:** 1 Center for Community Engagement and Impact Virginia Commonwealth University Richmond, VA United States; 2 L Douglas Wilder School of Government and Public Affairs Virginia Commonwealth University Richmond, VA United States; 3 Center on Society and Health Virginia Commonwealth University Richmond, VA United States; 4 Computer Science Department Virginia Commonwealth University Richmond, VA United States; 5 Department of Family Medicine and Population Health Virginia Commonwealth University Richmond, VA United States

**Keywords:** data augmentation, BERT, transformer-based models, text classification, community engagement, prototype, IRB research, community-engaged research, participatory research, deep learning

## Abstract

**Background:**

Community-engaged research (CEnR) is a research approach in which scholars partner with community organizations or individuals with whom they share an interest in the study topic, typically with the goal of supporting that community’s well-being. CEnR is well-established in numerous disciplines including the clinical and social sciences. However, universities experience challenges reporting comprehensive CEnR metrics, limiting the development of appropriate CEnR infrastructure and the advancement of relationships with communities, funders, and stakeholders.

**Objective:**

We propose a novel approach to identifying and categorizing community-engaged studies by applying attention-based deep learning models to human participants protocols that have been submitted to the university’s institutional review board (IRB).

**Methods:**

We manually classified a sample of 280 protocols submitted to the IRB using a 3- and 6-level CEnR heuristic. We then trained an attention-based bidirectional long short-term memory unit (Bi-LSTM) on the classified protocols and compared it to transformer models such as Bidirectional Encoder Representations From Transformers (BERT), Bio + Clinical BERT, and Cross-lingual Language Model–Robustly Optimized BERT Pre-training Approach (XLM-RoBERTa). We applied the best-performing models to the full sample of unlabeled IRB protocols submitted in the years 2013-2019 (n>6000).

**Results:**

Although transfer learning is superior, receiving a 0.9952 evaluation F1 score for all transformer models implemented compared to the attention-based Bi-LSTM (between 48%-80%), there were key issues with overfitting. This finding is consistent across several methodological adjustments: an augmented data set with and without cross-validation, an unaugmented data set with and without cross-validation, a 6-class CEnR spectrum, and a 3-class one.

**Conclusions:**

Transfer learning is a more viable method than the attention-based bidirectional-LSTM for differentiating small data sets characterized by the idiosyncrasies and variability of CEnR descriptions used by principal investigators in research protocols. Despite these issues involving overfitting, BERT and the other transformer models remarkably showed an understanding of our data unlike the attention-based Bi-LSTM model, promising a more realistic path toward solving this real-world application.

## Introduction

Transfer learning is widely used when comparing traditional machine learning and deep learning models [1]. It is likely that transformer models like Bidirectional Encoder Representations From Transformers (BERT) [2], a neural network-based technique for natural language processing (NLP) pretraining, will always play a substantial part in how we model language [3]. Researchers attempt to make use of these language models and fine-tune them to their classification tasks using various data sets. Superior results have been found with large data sets [4], small data sets [5,6], short text lengths [7], longer text lengths [8], and even data sets of different languages [1]. These studies, and the work reported here, demonstrate that better results can be achieved without substantial amounts of computing power and data.

Community-engaged research (CEnR) is a research approach in which investigators from conventional research institutions, such as universities, partner with community members or organizations with whom they share an interest, typically with the goal of advancing that community’s well-being [9]. Defined by its research philosophy and the relationship between research partners rather than methodology, CEnR is now an established scholarly tradition in numerous disciplines including health sciences, the social sciences, social work, urban planning, education, and the arts. Teams using CEnR have implemented research projects addressing a wide range of stakeholder concerns; collaborated with partners across the research process [10-13], from problem identification to scaling evidence-based interventions [14]; transformed service learning with new curricula and pedagogies that reflect students’ interests and learning styles [15]; and transformed natural, built, and artistic environments to better reflect the values and interests of communities [16].

CEnR’s flexibility and breadth has been productive, resulting in dedicated journals, conferences, courses, funding mechanisms, evaluation metrics, and theories of classification along continua of activities and structures of governance. Yet identifying, describing, measuring, and reporting on CEnR studies in the aggregate has been a challenge for universities and other institutions (eg, disciplinary associations [17]), in particular, reporting valid and reliable metrics to funders and stakeholders [17], and developing and maintaining appropriate internal CEnR infrastructure. Dependence on conventional review mechanisms such as scholarly databases to provide data on CEnR productivity may be limited by diversity in disciplines, methods, and dissemination approaches; impacts that are primarily shared outside of traditional scholarly mechanisms such as peer-reviewed journals; and inaccurate selection of CEnR as a keyword. The limited federal and foundation support available for CEnR obviates searches of funding databases. Moreover, established mechanisms for identifying and tracking CEnR may privilege recognition of CEnR collaborations that proceed along a unidirectional pathway in which relationships between professional researchers and community members demonstrate a deepening collaboration over time, resulting in grants and peer-reviewed publications. Such an emphasis both belies the reality of inequities in the distribution of resources needed to sustain such collaborations, for example, between disciplines, between research-productive and teaching institutions, and between established and junior faculty.

Virginia Commonwealth University (VCU) is an R01 institution designated by the Carnegie Foundation as “Community Engaged” with “Highest Research Activity.” In 2013, VCU began flagging CEnR studies using three custom fields [18] in the university’s online human participants protocol submission form, as part of an award from the National Center for Advancing Translational Sciences.

- Is there at least one community partner involved in the proposed study? (Yes/no answer)
- If yes, who is the community partner?
  Name of organization
  Zip code or country of the organization
- Which of the three statements below best describes the role of the community partner in the study?
  Community partners only provide access to study participants or project sites. They are not involved with study design, participant recruitment, data collection, or data analysis.
  Community partners do not make decisions about the study design or conduct but provide guidance to the researcher about the study design, participant recruitment, data collection, or data analysis.
  Community partners make decisions with the researchers about the study’s research activities or help conduct those activities (ie, study design, participant recruitment, data collection, or data analysis) [19].

Technical impediments to entering data into these custom fields were identified in 2018. This quality concern initiated a broader discussion among stakeholders across VCU about other possible limitations in the system of documentation, for example, inconsistent interpretation of these fields by principal investigators or study administrators submitting protocols. This discussion led to the exploratory study described here. The overall aim of this study was to develop a methodology to automatically detect CEnR studies among protocols submitted in the university’s online institutional review board (IRB) system, which contains data on all research with human participants [20]. This study provided the opportunity to test and build on the three custom fields added to the IRB protocol. The subaims are as follows: develop a system of classification to adapt the conventional theorization of CEnR across a spectrum of collaboration to the practical reality of studies conducted at an R01 university, determine if one or more deep learning models could automate the identification of CEnR studies trained by a subset of hand-labeled IRB protocols, and identify the best-performing algorithms and apply them to a retrospective 5-year data set of unlabeled research protocols (n>6000) that were not incorporated in the training of the algorithm.

## Methods

### Data

#### Data Collection

The first stage of this process was to pull research protocols from the IRB’s database (n>20,000). We then cleaned and deduplicated the records (1 study per protocol, “exempt,” “expedited,” “full,” and “started/submitted” protocols were included, but “not yet reviewed” studies were left out), leaving us with 6000 research studies, from which a sample (n=280) was randomly selected, reviewed, and manually labeled as one of the six classes (described in the Data Annotation section). Our criteria for selecting this sample set were based on a research study’s likelihood of being CEnR or not. Textbox 1 shows the chosen columns and a snippet of what the data looks like. Examples of the terminology we used for finding potential CEnR research are as follows: community-engaged, community-based participatory research, (community) action research, participatory action research, community advisory group, community steering, etc.

**The institutional review board protocol fields used to classify protocols with brief example sentences. These fields were concatenated into one column during training.**

**Study title**

- “Exploring dental service underutilization amon...”
- “Regional Scan and Strategies for Community Eng...”
- “Reflections on 5 years of community-based part...”

**Informed personnel**

- “The research team is in routine contact among...”
- “The team has three weekly meetings to inform t...”
- “We are a research team that collaborates on a...”

**Scientific benefit**

- “This research is intended to identify, describ...”
- “This study is meant to inform community leader...”
- “This study will address gaps in scientific know...”

**Aims and goals**

- “The overall aim of this mixed methods study is...”
- “Based on the results of the literature review,...”
- “The goal is to describe and publish the effect...”

**Identify participants**

- “ALL PARTICIPANTS Community Partner has experience admin...”
- “We will first scan regional organizational to...”
- “We already have contact and working relationsh...”

**Background**

- “Unmet dental needs are significant public heal...”
- “This project is part of a larger Richmond init...”
- “The field of CBPR still suffers from gap in e...”

**Hypothesis**

- “As a mixed-methods study, this research uses a...”
- “This project is to complete a literature revie...”
- “We are trying to document the direct and indir...”

**Study design**

- “STUDY DESIGNThis mixed methods study is a cros...”
- “Regional ScanFor the regional scan, the projec...”
- “We will talk to selected community partners an...”

#### Data Annotation

We uploaded the newly extracted sample data set into Google Sheets to facilitate a collaborative process of manually reviewing and labeling the protocols for use in training the algorithm. The team of three reviewers (two per research study) reviewed the available data for each protocol and labeled it “yes” (CEnR) or “no” (not CEnR) and assigned a class corresponding to the CEnR level (0-6). Protocols that did not receive the same designation by both reviewers were discussed and resolved in weekly meetings.

#### CEnR Levels

After a preliminary review of the protocols, the reviewers inductively developed a coding system to reflect the types of relationships described in the protocols. Textbox 2 shows a breakdown of CEnR levels that were used by reviewers.

**CEnR levels that were used to manually classify the training data.**

**No community-engaged research (CEnR; 0)**

Research without a partnership or community engagement

**Non-CEnR partnership (1)**

There is reference to a partnership, but the relationship is uncategorizable (eg, not adequately described) or not a traditional community-engaged partnership (eg, contractual relationships).

**Instrumental partnership (2)**

The community partner primarily facilitates access to the “inputs” needed to conduct the study (eg, posting recruitment flyers, providing participant contact information, extracting data, or providing study sites for observation).

**Academic-led partnership (3)**

Minimal yet important interaction between the research team and the community partner, which is often essential to project success (eg, academic partners take the lead on study design and research activities, with community partner involvement at particular points, such as troubleshooting recruitment or facilitating community meetings)

**Cooperative partnership (4)**

Shared investment and mutual consideration between the research team and the community partner, without shared decision-making (eg, community advisory boards that provided input on study design methodology, reviewed data collection instruments, interpreted findings, or informed dissemination plans)

**Reciprocal partnership (5)**

Community partners and research teams share decision-making power and governance (eg, community-based participatory research, team science, or steering committees with decision-making power).

#### Data Cleaning

After reviewing and classifying the protocols, we checked again for duplications, did manual spell-checking, and trimmed white space and any irrelevant symbols. Final data cleaning was completed with Python using the NLTK package (stop words, lemmatization, lowercase, removing punctuation, splitting contractions, and other RegEx operations).

#### Data Augmentation

We tested whether data augmentation techniques [21] (replacing and inserting words [22]) using the nlpaug library [23] to synthetically increase the amount of training data using DistilBERT [24] would improve the performance. Table 1 shows the number of samples before and after augmentation.

**Table 1:** Number of examples per class before and after data augmentation (second data set).

Class	Samples (before), n	Samples (after), n
0	82	1931
1	40	1427
2	11	1413
3	101	1564
4	32	1431
5	13	1404

#### Data Sets

We used three data sets: (1) the original sample of 280 hand-classified protocols, (2) an augmented data set of the 280 protocol expanded to 9170 samples using DistilBERT, and (3) versions of the first two data sets with 6 classes merged into 3. We tested the data set with fewer categories of CEnR to explore whether using broader categories would improve generalization of the models and prediction score. For data sets containing three classes, we collapsed 1s and 2s (=1); collapsed 3s, 4s, and 5s (=2); and kept the class 0 as is.

### Models

We explored four models to classify the data into the CEnR classes: bidirectional long short-term memory unit (Bi-LSTM), BERT, Bio + Clinical BERT, and Cross-lingual Language Model–Robustly Optimized BERT Pre-training Approach (XLM-RoBERTa) transformer models. We present model architectures and hyperparameters in this section.

#### Bi-LSTM Attention Model

**Figure 1:**
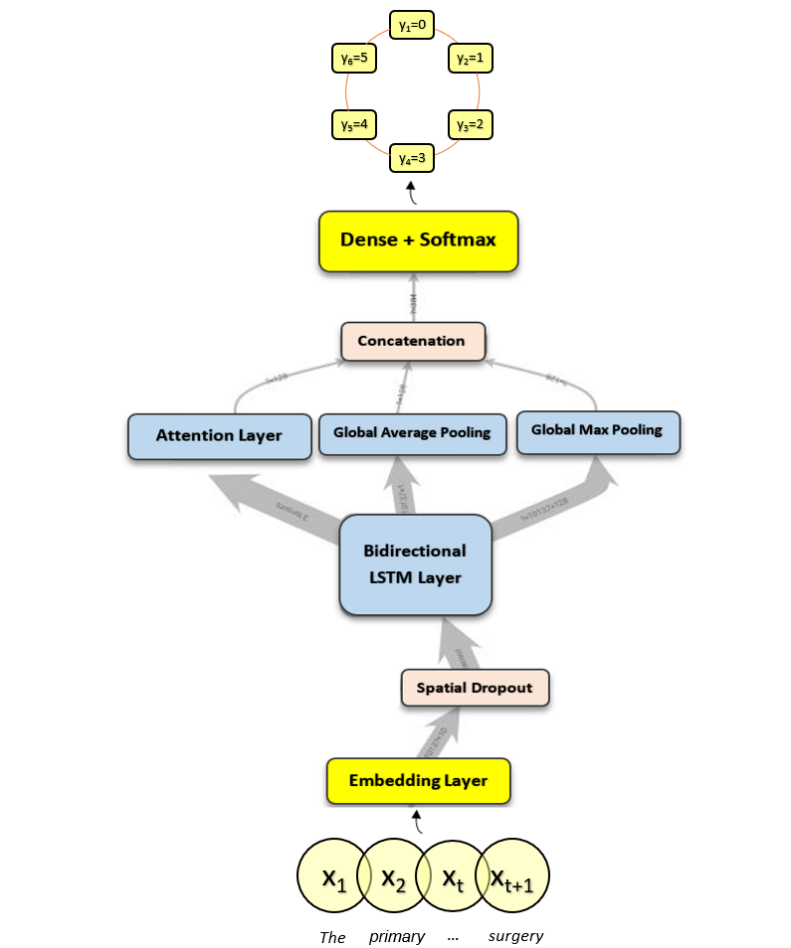
Attention-based bidirectional LSTM model architecture. LSTM: long short-term memory unit.

**Figure 2:**
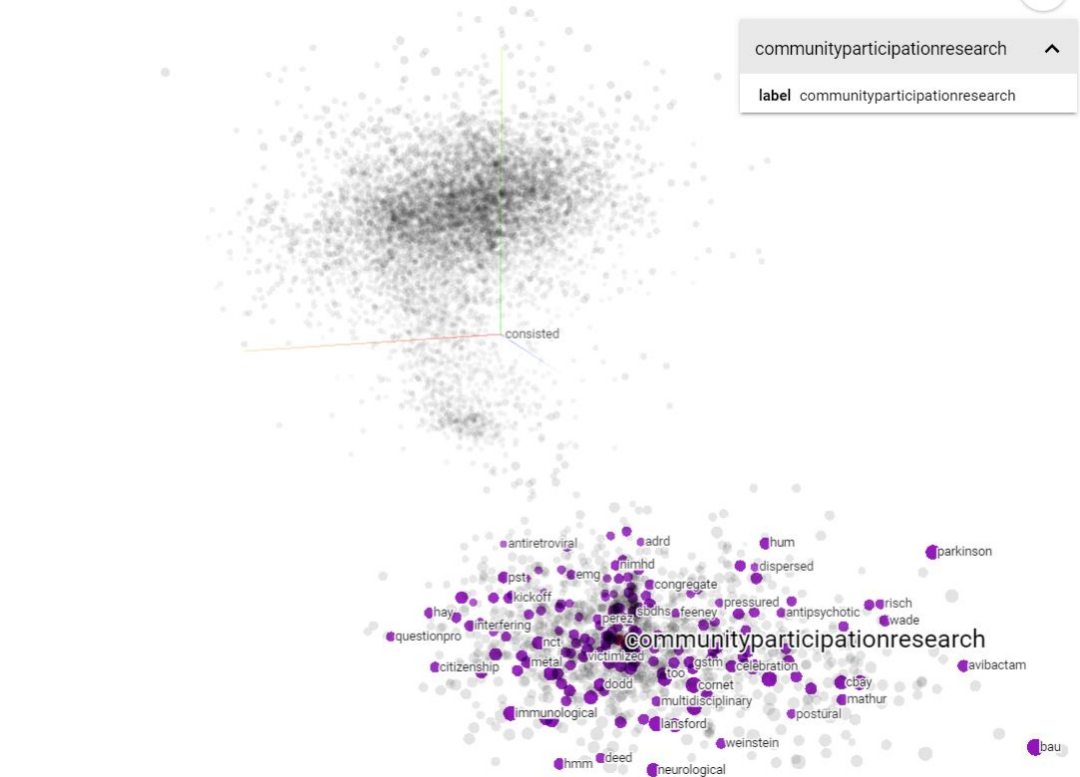
Searched “community participation research” in Google Embedding Projector.

Figure 1 illustrates the first model: a Bi-LSTM [25-27] with a basic custom attention layer [28,29] that was concatenated with a GlobalMaxPooling and GlobalAveragePooling layer. The embeddings used were the 100-dim Global Vectors for Word Representation (GloVe) embeddings file containing 400,000 words computed on a 2014 dump of English Wikipedia [30]. GloVe is an unsupervised learning algorithm for retrieving vector representations of words that can be plotted in a geometric space [31], as seen in Figure 2.

**Figure 3:**
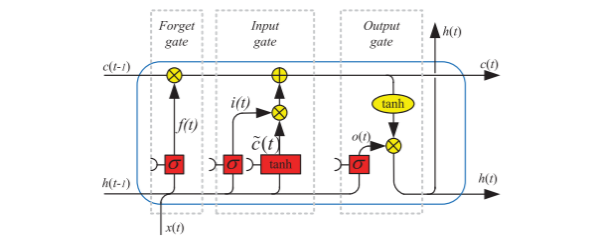
Architecture of long short-term memory unit.

The embedding layer captures the similarities between the words to best optimize for our inputs, and the Bi-LSTM runs through the data from the beginning of a sentence to the end and vice versa. This is done through its four [32] components as seen in Figure 3: cell state (*C_t_*), forget gate (*f_t_*), input gate (*i_t_* and 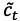), and output gate (*O_t_* and *h_t_*). These control the flow of sequential information, regulating what is important and what is not from those embeddings. The attention layer (which adds a weight of importance [33] to those Bi-LSTM outputs), the max pooling layer (which finds the most important features from the Bi-LSTM outputs), and the average pooling layer (which weighs all outputs from the Bi-LSTM as important) become fused together into one matrix to give the neural network more features to base predictions on. Finally, a dense layer with the softmax function is the flow of calculations made to give us a final output of a Y=[0,1,2,3,4,5] classification.

Stratified 7-fold cross-validation, Synthetic Minority Oversampling Technique (SMOTE) [34], and F1 macro optimization [35] were also used. Stratified K-fold cross-validation ensures the distribution of classes remains the same in every fold. SMOTE is a way to create fake data for the minority classes using examples that are similar (k-nearest neighbors). This technique was used within folds of cross-validation during training, not before. F1 macro optimization ensures that the F1 score is optimized during training, not accuracy. F1 macro refers to the average of the class’s F1 scores; this technique increased our evaluation F1 score by 7%.

#### Transformer Models

Transfer learning takes large and powerfully built language models that are pretrained on large corpuses of unlabeled data to later be fine-tuned and repurposed for a second related task, which can be beneficial for small data sets. A main aspect of this study was to see if the use of transfer learning improved the predictive performance for our text classification task. We used BERT-base-uncased [2], Bio + Clinical BERT [36], and XLM-RoBERTa [37] models, and tried different learning rates, batch sizes, and epochs for all three separately (around 30-50 different models trained per transformer). The Results section shows the best-tuned model for each transformer.

##### Bidirectional Encoder Representations From Transformers

Our first approach to transfer learning was fine-tuning the pretrained BERT model for our text classification problem. BERT was introduced by Devlin et al [2]. It was pretrained on BookCorpus (800 million words) and Wikipedia (2500 million words). The model’s architecture ensures its advantage in NLP tasks because it learns the contextual meanings of words and how each word is being used in a sequence due to its 12 attention heads and 110 million total parameters. GloVe embeddings do not consider the context of how a word is used and do not capture the different semantics that words can have (eg, a bat can be an animal or baseball equipment); thus the word “community” or “partner” can be used differently across different research studies. BERT, however, would capture those differences. Additionally, BERT can achieve state-of-the-art results on various tasks for large and small data sets, and it does not need to be trained for more than 2 to 4 epochs.

##### BIO + Clinical BERT

The second approach to transfer learning is fine-tuning with Bio + Clinical BERT [36]. As mentioned previously, BERT is pretrained on BookCorpus and Wikipedia, and in general can model language well for any NLP task; however, Alsentzer et al [36] examined ways to improve the general language model in BERT using BERT models geared for clinical text and discharge summaries. They demonstrated that performance is improved with domain-specific pretrainings, which is distinct from general language. The authors used data from the MIMIC-III database in two ways, clinical BERT (contains all note types) and discharge summary BERT (only contains discharge summaries), to further downstream tasks with clinical data that can be used for more specific classification problems. They then trained two BERT models on the clinical text, where one is initialized from the BERT-base model and the other was initialized from BioBERT (the model we chose).

##### Cross-lingual Language Model–Robustly Optimized BERT Pre-training Approach

Our third approach to transfer learning was an interesting model to fine-tune, mainly because this type of transformer model was not created for our kind of task; however, it still performed well. It was introduced by Conneau et al [37] in 2019 and updated in 2020. This model closely resembles the RoBERTa architecture [38], except it is a cross-lingual model pretrained on 100 different languages. This type of model is made for cross-lingual transfer learning tasks trained on more than 2 terabytes of the CommonCrawl corpora.

#### Other Models

Other models were used for this study, such as convolutional neural networks (CNNs), deep neural networks (DNNs), CNN + LSTM, CNN + Bi-LSTM, CNN + Bi-LSTM with attention, CNN + LSTM with attention, CNN + gated recurrent unit (GRU), CNN + Bi-GRU, CNN + Bi-GRU with attention, and CNN + GRU with attention; however, they did not perform as well as the Bi-LSTM + attention (ranging from a 0.30-0.40 evaluation F1 scores); therefore, we did not include their results in this paper.

### Experimental Details

#### Bi-LSTM Attention Model

In this model, we used the Keras libraries for training, tokenizing, and padding the sequences of text. The Bi-LSTM model was trained for 40 epochs, had a learning rate of 0.001, batch size of 64, and was trained for 12 hours; additionally, we used the Adam optimizer and sparse categorical cross entropy for our loss. The max sequence length after cleaning the data was 10,137. The model was trained as a CuDNNLSTM, which is a faster implementation of the LSTM backed up by CuDNN, which can only be run on a GPU.

#### Transformer Models

We used the SimpleTransformers library created by Rajapakse [39], which can train and evaluate transformer models (derived from the HuggingFace web site) with few lines of code. The hyperparameters for each transformer model can be seen from a web site called Weights and Biases that organizes and captures all the necessary data during training [40,41]. Since the text field lengths in our sample were longer than the limits for BERT and other transformer models, we used a sliding window technique. Here, any sequence from the data that exceeds the maximum sequence length will be split into several subsets, each pertaining to the length of the max sequence length value. Using this technique, each subset from the sliding window has overlapping values, also referred to as the stride (stride 0.8) resulting in about a 20% overlap between the windows. This process lengthens training time but is preferable to truncating data during training. All models were trained using Google Colab Pro and had weights corresponding to a class so that it was equally balanced during the training [42].

### Evaluation Metrics

The models trained were evaluated using the F1 score macro, which takes a balanced measure of precision and recall, and then the average of the F1 scores.

## Results

Table 2 shows the holdout F1 scores for each of our models on our original and augmented data sets with and without cross-validation. The evaluation F1 scores (not shown in the table) for the Bi-LSTM averaged 63.25%. From the order of Table 2, it was 65% (with cross-validation, augmented) and 48% (without cross-validation, augmented) for 6 classes, and 80% (with cross-validation, augmented) and 60% (without cross-validation, augmented) for 3 classes, whereas the transformer model’s evaluation F1 scores were all over 99%. We used Bio + Clinical BERT because domain-specific pretrainings have been shown to improve performance [34], and because our data set contains clinical research data, we thought it was relevant to compare its results. XLM-RoBERTa proved to do well and had an overall great understanding of the data, so it was included in this experiment as well. The holdout data set comprises 30 samples, which is almost too small to give an accurate account of how the models do, so our team will be working on labeling additional data. It is also a bit deceptive with the results shown because the classifications for the Bi-LSTM attention model were way off, whereas when the transformer models misclassified a research study, it was off by 1 or 2 classes. A lot of the results are not shown in the table. This is because it was not worth training the original data set without cross-validation due to the data set’s size, which would also make the evaluation data set different, and there was no training for Bio + Clinical BERT and XLM-RoBERTa for augmented data sets using cross-validation due to computational limitations.

**Table 2:** Results of the various models over the original and augmented data sets.

Model	Data	6 classes, F1 scores		3 classes, F1 scores	
		With CV^a^	Without CV	With CV	Without CV
Bi-LSTM^b^ w/ attention	Original	0.2000	N/A^c^	0.3000	N/A
Bi-LSTM w/ attention	Augmented	0.2667	0.3000	0.4000	0.2667
BERT^d^-base uncased	Original	0.2333	N/A	0.5000	N/A
BERT-base uncased	Augmented	0.3333	0.4000	0.4667	0.5333
Bio + Clinical BERT	Original	0.3000	N/A	0.4667	N/A
Bio + Clinical BERT	Augmented	N/A	0.4000	N/A	0.4333
XLM-RoBERTa^e^	Original	0.3667	N/A	0.4667	N/A
XLM-RoBERTa	Augmented	N/A	0.4000	N/A	0.4667

^a^CV: cross-validation.

^b^Bi-LSTM: bidirectional long short-term memory unit.

^c^N/A: not applicable.

^d^BERT: Bidirectional Encoder Representations From Transformers.

^e^XLM-ROBERTa: Cross-lingual Language Model–Robustly Optimized BERT Pre-training Approach.

## Discussion

### Principal Findings

The transformer models performed significantly better than the Bi-LSTM with attention. They were nearly perfect for their evaluation scores (all hitting 0.995) across all the data sets used (they overfit on the holdout data sets due to the same learning rate being used for each layer). Additionally, all models showed slight improvements when the number of classes fit a 3-class spectrum as opposed to a 6-class spectrum. It was hard to tell if the augmented data sets gave an advantage to the models; therefore, there is a need to research other techniques for that. Cross-validation for the Bi-LSTM significantly improved its results for the evaluation scores but that did not carry over into the holdout data sets. The best-performing models for the 6-class spectrum was a 3-way tie between the transformer models that did not use cross-validation. Cross-validation was not needed when using the augmented data sets in terms of their holdout set scores. Although the BERT model trained on the augmented data set without using cross-validation had superior performance (0.533 holdout F1 score), the second best-performing model (BERT trained on the original data set with cross-validation) with less data trained much faster, and the results differed only fractionally compared to the best-performing one. We believe that data augmentation has great potential (considering it gives more data), and it may confer advantages during a model’s training, but we feel it is better to go without it until more strategies are investigated. The strategies used were a faster way of synthetically creating more data, which does not necessarily mean it was the best way.

The Bi-LSTM attention model did not delineate between the classes nearly as well as BERT and the other transformer models, which has given our team a proof of concept, something to work with and improve on moving forward, whether that be more data or more computational power. Additionally, since there were only minor differences within the research study’s augmentations (simple replacing and inserting of contextual similar words), BERT and the other transformers were able to pick up on those patterns almost perfectly compared to the Bi-LSTM model.

This study demonstrates that transfer learning performed better for classifying levels of CEnR. However, the results for the holdout sets were still relatively low (highest was 0.533), which we hope to improve with an increased data set size. We were impressed by the efficiency of BERT and other transformer models. While it took months of testing to identify the approach for using the Bi-LSTM with attention, and even more time to tune the hyperparameters, in a single day, BERT was able to achieve performances like the results shown in Table 2, with a significant decrease in training time. Considering those advantages, transfer learning appears to come out on top when it comes to hyperparameter selection.

**Figure 4:**
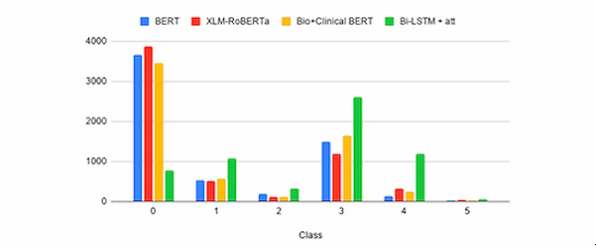
Model predictions on 6000 research studies. att: attention; BERT: Bidirectional Encoder Representations From Transformers; Bi-LSTM: bidirectional long short-term memory unit; XLM-ROBERTa: Cross-lingual Language Model–Robustly Optimized BERT Pre-training Approach.

The transformer model’s final predictions versus the Bi-LSTM’s final predictions on the remaining unlabeled data set are shown in Figure 4. The figure shows that predictions with the highest levels of engagement (4s and 5s) were lower from the transfer learning models, indicating a better understanding of our data in the real world, where 4s and 5s are infrequent in the data set and most protocols are zeros. This is the case because the IRB database represents all types of research, of which CEnR is a relatively small fraction. Bio + Clinical BERT and XLM-RoBERTa had results that were like BERT, although BERT was arguably more realistic. Of the transformer models, they agree on almost 4000 research studies’ predictions; however, the attention-based model is only in agreement with all of them 850 (of the 6000) times.

### Limitations

Researchers had the option of attaching detailed protocols as a PDF file instead of filling out the database fields. We were not able to retrieve PDF data for this study, reducing the total number of studies, which limited what data we could label. In addition, we observed that the transformer models predicted larger classes compared to smaller classes (eg, levels two, four, and five). Nevertheless, they still made reasonable predictions, which is exciting to see because it means we can improve from this issue moving forward by labeling more data or sticking to the 3-class spectrum. We were also limited in our ability to compute very large models when using Google Colab Pro, which has certain computing limitations. Another time-consuming step was reviewing and labeling the data. The transformer models were derived from a library in which the overall structure is in its basic form; therefore, more adjustments can be made on their architectures [4,8].

### Conclusions

In conclusion, we compared widely used techniques in classification tasks: transfer learning using BERT, Bio + Clinical BERT, XLM-RoBERTa, and a Bi-LSTM attention model. We found that transfer learning performed best for our purposes and was quick and easy to implement. Additional work is needed to apply the model in a system. In terms of process, we found that augmenting the data set has the potential to improve the results, cross-validation was not as helpful for the transformer models when using a less general classification spectrum, hyperparameter tuning with transformer models was less stressful and time-consuming, transformer models can handle small data sets well, and condensing the 6 classes into 3 was a less rigid spectrum for models to differentiate and provided superior results.

Additional improvements can be made, such as correcting a sample from the final prediction’s data set by using the same search word criteria as before (Data Collection section) or by taking a random sample to increase our training data. We could also use different augmentation techniques, as there are other ways this could have been implemented. Future work includes fine-tuning strategies and hyperparameter optimization such as discriminative learning rates, slanted triangular learning rates, and freezing layers. BERT is the best model from this study mainly because of its holdout score for the 3-class spectrum, and its training time is much faster than the other two transformer models; however, moving forward, all three transformer models will continue to be used in improving this experiment, as each is unique in their understanding of the data.

Identifying CEnR and classifying levels of engagement allow us to understand the types of research taking place across the university. These data can help organizations better serve their stakeholders and to plan for the infrastructure needed to support community engagement. Additionally, tracking these metrics can help institutions report to funders and stakeholders on their engagement activities. The innovative aspect of this methodological study is creating an automated system to categorize research using administrative data. This study describes how transformer models can automate this process.

## Abbreviations

BERT: Bidirectional Encoder Representations From Transformers
Bi-LSTM: bidirectional long short-term memory unit
CEnR: community-engaged research
CNN: convolutional neural network
DNN: deep neural network
GloVE: Global Vectors for Word Representation
IRB: institutional review board
NLP: natural language processing
SMOTE: Synthetic Minority Oversampling Technique
VCU: Virginia Commonwealth University
XLM-RoBERTa: Cross-lingual Language Model–Robustly Optimized BERT Pre-training Approach
